# Quality improvement in the undergraduate medical curriculum: the need for clinical exposure

**DOI:** 10.1186/s12909-021-02566-2

**Published:** 2021-09-01

**Authors:** Jemima Carter, Suzanne Capon, Claire Dyer, Maya Whittaker

**Affiliations:** Kings College London, GKT School of Medical Education, London, UK

**Keywords:** Quality improvement, Patient safety, Medical education

## Abstract

This correspondence article aims to outline the importance of an integrated clinical component within Quality Improvement education in response to the recently published article by Shah et al.. The Quality Improvement and Patient Safety workshops described in the above study were compared with the Quality Improvement module experienced by medical students at King’s College London. The key difference between the two methods of teaching Quality Improvement was the clinical project undertaken by King’s College Students, which helped students gain an appreciation of the pitfalls of instigating change in a clinical environment. The authors feel that this arguably more authentic experience could have benefited the students in the study in making them feel better equipped to use the skills learned in the theoretical workshops in their later careers.

## Main text

Dear Editor,

We read with interest the article by Shah et al. describing a pilot study of quality improvement and patient safety (QI/PS) workshops for students [1]. As final year medical students at King’s College London, we have personal experience of the benefits and pitfalls of undertaking a mandatory QI/PS module. We are keen to compare the different ways in which a QI/PS education is delivered by our respective institutions. Understanding and applying principles of QI/PS forms part of the GMC Outcomes for Graduates, a list of required skills for newly qualified doctors in the United Kingdom, yet there is no specific guidance around how best to incorporate this into the undergraduate medical curriculum [2].

The use of simulated workshops as described by Shah et al. would help students to develop a theoretical understanding of QI/PS, however we put it to the authors that actioning these techniques in clinical environments can be far more complex. In contrast to the QI/PS teaching described, our first experience of QI/PS was directly designing and undertaking a QI project in a clinical setting. This experience showed us the realistic barriers of QI/PS; didactic teaching and simulated workshops would not have fully addressed clinical issues such as time management, health professionals’ engagement, incentive to change and bureaucracy. We see the additional benefit that experiencing these challenges for oneself would have in preparing students for undertaking such projects in their future careers.

Comparably, our QI module lacked the didactic teaching described by Shah et al. Our appreciation for the theoretical aspect of QI could have been greatly enhanced if our institution had incorporated this style of teaching.

The teaching model adopted by Shah et al. whereby students designed QI/PS workshops and delivered these to their peers could raise questions regarding the quality of the teaching. Our QI project was always under the guidance of a faculty member with a background in QI. Although we appreciate that a proportion of the workshops in the pilot study had staff facilitators, we believe that all workshops should be supervised by a faculty member with experience in QI, as this would provide the added benefit of discussion of the real-life challenges, as mentioned above. Research suggests that peer-led teaching is effective in engaging students, however one study found that 85.1 % of students benefited from expert teachers summarising the topic at the end of such sessions, implying that expert supervision is important for the success of these student-lead workshops [3].

Given that the foundation of medicine should be evidence-based practice, we believe QI/PS teaching should be a mandatory part of medical education, as supported by the GMC guidelines. Implementing clinical QI/PS projects under the supervision of experienced clinicians as part of the undergraduate curriculum should be achievable for medical schools as practising clinical teachers should have experience QI/PS as per GMC guidance [2]. Although the participant responses for the workshops devised by Shah et al. were overwhelmingly positive, there was a potential issue with participation bias, as the workshops were attended by students “with a genuine interest”. If the programme was rolled out on a mandatory basis, students’ desire to engage with the workshops may have been more varied, which may have been reflected in the students’ feedback. Research into medical students’ attitudes to QI education supports that clinical integration is highly preferable to didactic teaching alone, and if the pilot study were to be expanded as a mandatory module, adding an authentic clinical component, such as partaking in an audit under supervision, may enhance student satisfaction [4].

## Data Availability

Not applicable.

## Abbreviations

QI: Quality Improvement
PS: Patient Safety
GMC: General Medical Council
